# Characterization of Water Structure and Phase Behavior within Metal–Organic Nanotubes

**DOI:** 10.1021/acs.langmuir.3c02786

**Published:** 2023-12-11

**Authors:** Tiron H. L. Jahinge, Maurice K. Payne, Daniel K. Unruh, Ashini S. Jayasinghe, Ping Yu, Tori Z. Forbes

**Affiliations:** † Department of Chemistry, University of Iowa, Iowa City, Iowa 52242, United States; ‡ Nuclear Magnetic Resonance Facility, University of California, Davis, Davis, California 95616, United States

## Abstract

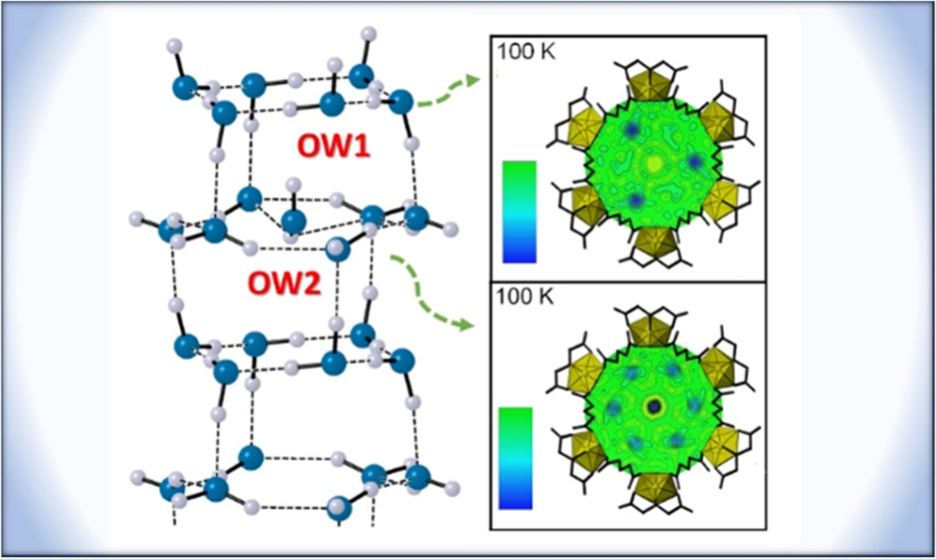

Water behavior under nanoconfinement varies significantly from that in the bulk but also depends on the nature of the pore walls. Hybrid compound offers the ideal system to explore water behavior in complex materials, so a model metal–organic nanotube (UMONT) material was utilized to explore the behavior of water between 100 and 293 K. Single-crystal X-ray and neutron diffraction revealed the formation of a filled Ice-I arrangement that was previously predicted to only occur under high pressures. ^17^O NMR spectra suggest that the onset of melting for the water in the UMONT channels occurs at 98 K and the presence of ice-like water up to 293 K, indicating that the complete ice–water transition does not occur before dehydration of the material. Overall, the water behavior differs significantly from hydrophobic single-walled carbon nanotubes indicating precise control over water can be achieved through rational design of hybrid materials.

## Introduction

Water confined within nanoscale pores has previously been reported to differ from bulk phase behavior,^1−5^ dynamics,^3,6−10^ and structure,^2,11,12^ and these changes are critical to processes in both natural and engineered systems. Within natural biological systems, such as AQP1 aquaporin channels, nanoconfined water molecules are arranged in unique one-dimensional (1D) water wires that enable precise mobility and exhibit fast transport rates (3 × 10^9^ s^–1^ per channel).^13−15^ Water is also trapped in the nanoscale pore spaces and channels of geologic materials, such as zeolites,^6,16,17^ clay minerals,^18,19^ or sedimentary rocks that result in variable melting point temperatures, density, and surface tension and impact transport behavior.^20^ Within engineered systems, behavior of nanoconfined water has been explored in mesoporous silica,^9,21,22^ carbon nanotubes,^23−34^ graphene sheets,^23,26,35^ inorganic nanotubes,^36−39^ and lipids,^12,40^ with all systems exhibiting behavior that diverges from the bulk. These chemical and physical changes are likely dependent on both the size of the pore space and the chemical character of the interior walls. However, additional investigations on model systems would enable the development of precise structure–function relationships that would link the physiochemical properties of the pore walls and the overall behavior of nanoconfined water molecules.

Prior work exploring these structure–function relationships has mainly focused on either purely hydrophobic or hydrophilic nano porous materials, but hybrid compounds may offer the ability to understand the behavior of nanoconfined water in more complex systems. In the case of hydrophobic pores, carbon-based materials, namely, single-walled carbon nanotubes, have offered the most insight into the system. Due to the repulsion between the interior channel walls and encapsulated water molecules, hydrophobic carbon nanotubes strongly encourage hydrogen-bonding interactions between the nanoconfined water molecules.^23,26−28,41^ This leads to ordered arrangements of water molecules that have similarities to ice-like arrays, with structural variability occurring due to the overall pore size.^29−31,33^ In addition, spontaneous uptake of water and fast mass transport have been reported for these systems because the extended arrays of hydrogen-bonding water networks do not interact strongly with the pore walls.^25,26,28,42^ Strictly hydrophilic pores exhibit strong interactions between the confined water molecules and hydrophilic groups (such as hydroxyl) and can create islands of highly coordinated local water regions that are also more ordered than that of bulk water.^20,37−39^ In addition, the liquid–solid phase transition is depressed in hydrophilic environments, with smaller pore sizes (1.5 nm) leading to melting/freezing temperature changes of up to 40 K.^20^ Hybrid materials offer an interesting middle ground that can provide variability in these behaviors. Materials that possess both hydrophilic and hydrophobic surface groups within the pore walls will engage with water molecules in complex ways, which can then further alter the behavior of nanoconfined water molecules.^43−45^ The exact placement of these functional groups provides precisely defined attractive and repulsive forces and advanced control of water orientation, diffusion, and selectivity that is yet to be observed within pure hydrophobic or hydrophilic systems.^43^

To further understand the impacts of more complex hybrid nanoconfinement environments, we can utilize metal–organic materials as a model system. Metal–organic materials, such as metal–organic frameworks or metal–organic nanotubes, contain a metal node connected through organic linkers that then create the basis for the pore walls.^46−48^ These materials are known for creating uniform pore spaces and offer design flexibility because the organic ligands can be modified to create subtle differences in the hydrophilic or hydrophobic nature of the molecules.^49^ In addition, the materials form extended crystalline networks that enable the use of single-crystal X-ray diffraction techniques to clearly evaluate the water structure and ordering.

We have previously reported a metal–organic nanotube (UMONT) that displays unique water properties and can serve as a model system to further evaluate the structure and dynamics nanoconfinement of water in a hybrid environment.^50−54^ In the current study, we explore the temperature-dependent behavior of the water within the UMONT material using diffraction, NMR spectroscopy, and differential scanning calorimetry (DSC) to further evaluate the structure and dynamics of confined water within mixed hydrophobic–hydrophilic systems. These results were then compared to previous work on pure hydrophobic and hydrophilic pore walls to determine the divergent behavior of nanoconfined water within hybrid materials.

## Experimental Section

### Synthesis of UMONT

Single crystals of the UMONT compound were synthesized according to previously published methodology.^50^ Briefly, uranyl nitrate hexahydrate (2.5 mL of 0.2 M) and iminodiacetic acid (5 mL of 0.2 M) were mixed in a glass vial, followed by an additional 5 mL of 0.2 M piperazine. *CAUTION: ^238^U is a radioactive element and is handled by trained personnel in a licensed facility.* The solution was mixed, and then methanol or acetone was added to the solution in a 1:1 vol ratio to aid in the crystallization. The vial was capped, and UMONT crystals formed within 3 days at 95% yields. For neutron diffraction experiments, the synthesis was repeated by using deuterated compounds when possible. Deuterated piperazine and methanol were purchased from Sigma-Aldrich, whereas deuteration of the iminodiacetate and uranyl nitrate hexahydrate occurred through exchange and recrystallization of the solid phase with 99.9% D_2_O. Deuteration of the reactants was confirmed by NMR spectroscopy. Crystallization of the solid material also occurred by using 99.9% D_2_O as the solution.

### Variable-Temperature Single-Crystal X-ray Diffraction

To confirm the hydration of the UMONT material before structural characterization, the crystals were placed in a small vial and exposed to a saturated (RH = 80%) environment. High-quality single crystals were isolated, coated in oil, and mounted on a Bruker D8 Quest CCD single-crystal X-ray diffractometer equipped with Mo Kα radiation (λ = 0.7107 Å) and a low-temperature cryostat (Oxford Cryosystems, Cryostream 800). Initial data were collected at 100 K with the APEX III software, and then variable-temperature studies were performed on single crystals of both the hydrated and dehydrated forms. Data was collected at each temperature and a dwell time of 15 min before data collection allowed the material to reach the desired temperature value. X-ray diffraction data was collected from 100 to 295 K at 50 K increments, but the region between 200 and 260 K was also collected at 10 K increments. Previous results indicated that the UMONT material begins losing water at 295 K, with complete dehydration occurring at 335 K. Thus, 295 K was the maximum temperature at which X-ray diffraction data was collected for this study.^50^ The experiments were repeated to ensure that the data was consistent.

All diffraction data were integrated, and then peak intensities were corrected for Lorentz, polarization, and background effects using the Bruker APEX III software. An empirical absorption correction was applied using the program SCALE and the structure solution was determined by intrinsic phasing methods and refined on the basis of *F*^2^ for all unique data using the SHELXTL (version 5)^55^ within the OLEX2 software suite. For the first data set, U atoms were located by direct methods, and the O, N, and C atom positions were identified in the difference Fourier maps calculated following refinement of the partial-structure models. Hydrogen atom positions associated with the organic linkers were fixed using a riding model, whereas the H atoms for water molecules were identified in the difference Fourier maps when possible. This final structural model was used as the basis for the other data collected at different temperatures to ensure consistency in the orientation and position of the unit cell.

Electron density maps of the water molecules within the UMONT nanotubes were generated by OLEX2 software suit.^56^ These maps were created after finalizing structural refinement of the uranyl iminodiacetate and piperazinium components but without modeling the water molecules within the nano porous channels. This asymmetric molecular unit was expanded to a full symmetry molecular unit using the “grow” command and oriented so that the [001] direction was visible using the “matr 3” command. The contour plus plane mode for the electron density map was chosen for the visualization of the residual electron density within this region. The residual electron density maps provided in this study are generated by adjusting the map size to 8, the number of contours to 15, and the colors to 5. Specific different depth values (−1, and −7) were chosen because they were associated with the planes that represented the OW1 and OW2 sites. Scaling of the electron density maps was initially performed in the automatic option as it provided the minimum electron density that is presented in the selected plane.

### Neutron Diffraction

Neutron scattering measurements were conducted by measuring a single crystal (dimensions 0.60 × 0.60 × 1.0 mm^3^) in the time-of-flight single-crystal Laue diffractometer TOPAZ at Oak Ridge National Laboratory. Sample orientations were optimized with the CrystalPlan software.^57^ Reduction of the raw data including Lorentz corrections, absorption, time-of-flight spectrum, and detector efficiency corrections were carried out with ANVRED3.^58^ The raw peaks were integrated using a three-dimensional (3D) ellipsoidal routine,^59^ and the reduced data set was refined using SHELXL.^55^

### NMR Experiments

The UMONT material was heated to 393 K to remove the nanoconfined water and then rehydrated in a saturated environment containing ^17^O labeled water. All solid-state NMR experiments were performed on a Bruker AVANCE 500 solid-state NMR spectrometer equipped with an 11.74 T magnet and a Bruker 4 mm MAS probe. The resonance frequency was 67.8 MHz for ^17^O. Room temperature ^17^O magic angle spinning (MAS) NMR experiment was performed by employing a single 30° pulse sequence with a recycle delay time of 0.1 s and 15 kHz of spinning speed. The data were averaged over 20,548 scans and processed with 100 Hz of exponential line broadening. Static ^17^O NMR data were collected by using a Hahn Echo pulse sequence with a solid 90° pulse length of 1.37 μs. The interpulse delay time was varied from 14 to 24 μs, and 22 μs was optimal for echo formation and signal intensity. The recycle delay time was also varied from 0.05 to 1 s, but there was no significant difference of the signal intensities between the qualities of 0.1 and 1 s. The final parameters used for data acquisition were 0.1 s of recycle delay time and 22 μs of interpulse delay time. Static ^17^O NMR data were collected in the temperature range of 293–193 K and temperatures were calibrated by ^207^Pb NMR spectra.^60^ The chemical shift was referenced to tap water (0 ppm) for the ^17^O NMR.

### Differential Scanning Calorimetry

DSC analysis of the hydrated UMONT samples was conducted using a TA Instruments Q500 differential scanning calorimeter, equipped with a mass flow controller. Approximately 10–20 mg of the UMONT sample was loaded into a preweighed aluminum pan and hermetically sealed before loading onto the instrument. The data was collected from 100 to 360 K at a ramp rate 5°/min and analyzed using the TA Instruments TRIOS software.

## Results and Discussion

### Structural Characterization Using X-ray Diffraction

For the variable-temperature study, a single crystal of hydrated UMONT was evaluated using a full structural analysis at each of the chosen temperatures, and the data parameters for seven different points (100, 195, 210, 220, 230, 250, and 270 K) are summarized in the Supporting Information. The unit cell dimensions remain relatively constant through the temperature regime with the parameter increasing from 22.2935(7) Å at 100 K to 22.5614(9) Å at 250 K. We note that there is a small decrease in the parameter when the temperature is increased to 270 K as the value becomes 22.5295(11) Å. For the *c* parameter, the change is relatively constant with the value at 100 K starting at 6.6090(3) Å and the largest change was noted as 0.0244 Å. The crystallinity and high-quality data collection were also confirmed, as the *R*_1_ value ranged from 3.53 to 4.57% for all data sets when the two crystallographically unique water molecules (OW1 and OW2) in the UMON channel are included in the model.

From our previous work on the thermal expansion behavior of the UMONT material, we noted similarities in the unit cell parameters over this temperature range. The prior temperature study was performed with no additional dwell time, but we also noted the *c* parameter remains relatively constant and then decreases by 0.05 Å as the temperature increases from 200 to 260 K.^55^ This former study also reported that the parameter increased slightly between 100 and 200 K and then more substantial gains between 200 and 260 K. Since the previous work by Payne et al.^61^ only evaluated the unit cell and thermal expansion parameters, we needed additional analysis to evaluate the structural and dynamic changes for the nanoconfined water in the UMONT channels.

**Figure 1:**
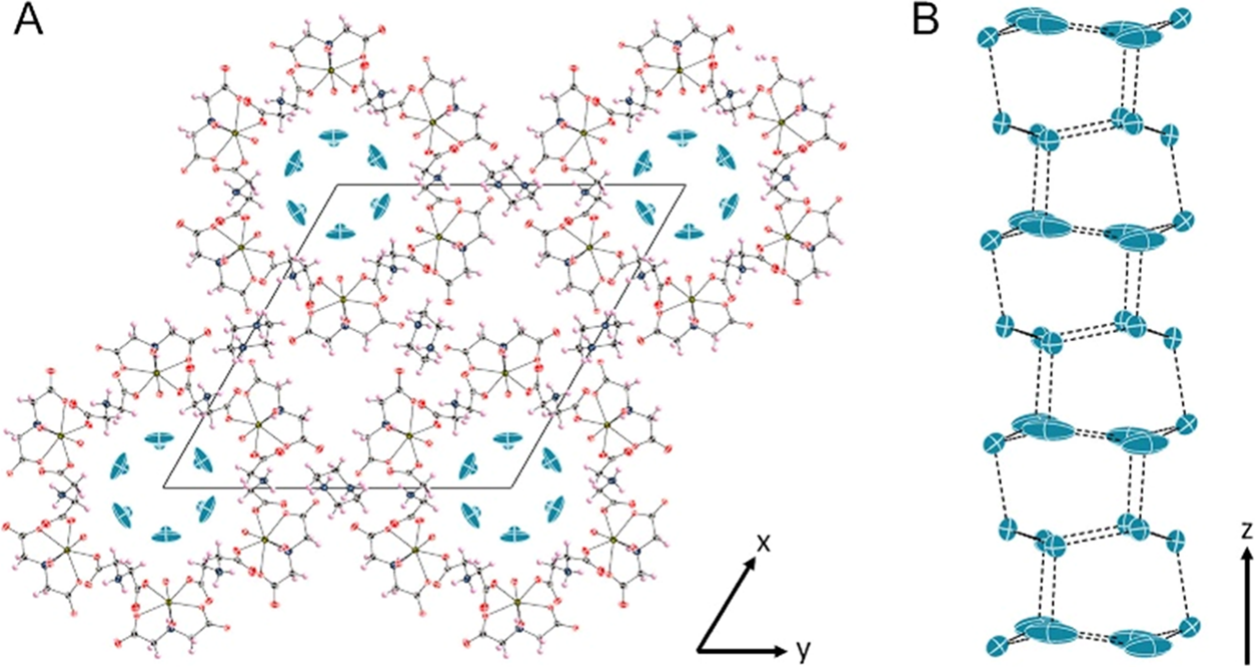
(A) UMONT compound contains U(VI) cations coordinated by iminodiacetate ligands to create a metal–organic nanotube with a diameter of 1.18 nm diameter. The U, C, N, O, and H atoms associated with the nanotubular arrays are depicted as yellow, black, light blue, red, and pink ellipsoids, respectively. Water molecules (teal ellipsoids) are located as a hexagonal water array down the (B) length of the UMONT channel. The hydrogen atoms associated with the nanoconfined water are not shown for clarity.

The general structure of the UMONT material includes nanotubular arrays composed of U(VI) metal nodes connected through iminodiacetate linkers (Figure 1a). Each of the U(VI) metal centers is strongly bound to two oxygen atoms in the axial positions to create the uranyl (UO_2_)^2+^ cation. Iminodiacetate molecules further coordinate in the equatorial plane through both tridentate chelation and monodentate linkages to connect the UO_2_^2+^ metal centers into a six-membered macrocyclic unit. Further hydrogen bonding occurs between the macrocycles to create the nanotubular arrays that are arranged into a highly crystalline 3D lattice through interactions with piperazinium counterions. The macrocycles have a 1.18 nm pore space that contains water molecules that are arranged in six-membered rings to create a hexagonal ice-like array along the [001] axis.

Our initial structural characterization of UMONT at 100 K suggested two crystallographically unique water molecules (OW1 and OW2) occupy the nanopores.^50^ Symmetry within the unit cell results in the formation of hexameric rings of water for the OW1 and OW2 molecules that run the length of the UMONT channel (Figure 1b). Thermal ellipsoid representation at 100 K suggests that the OW1 site is more ordered and occurs in chair confirmation. The OW2 site has larger thermal ellipsoids, suggesting more positional disorder at this site. Donor-to-acceptor bond distances for hydrogen bonding within the channel are 2.810, 2.850, and 3.179 Å for OW1-OW1, OW1-OW2, and OW2-OW2, respectively.

**Figure 2:**
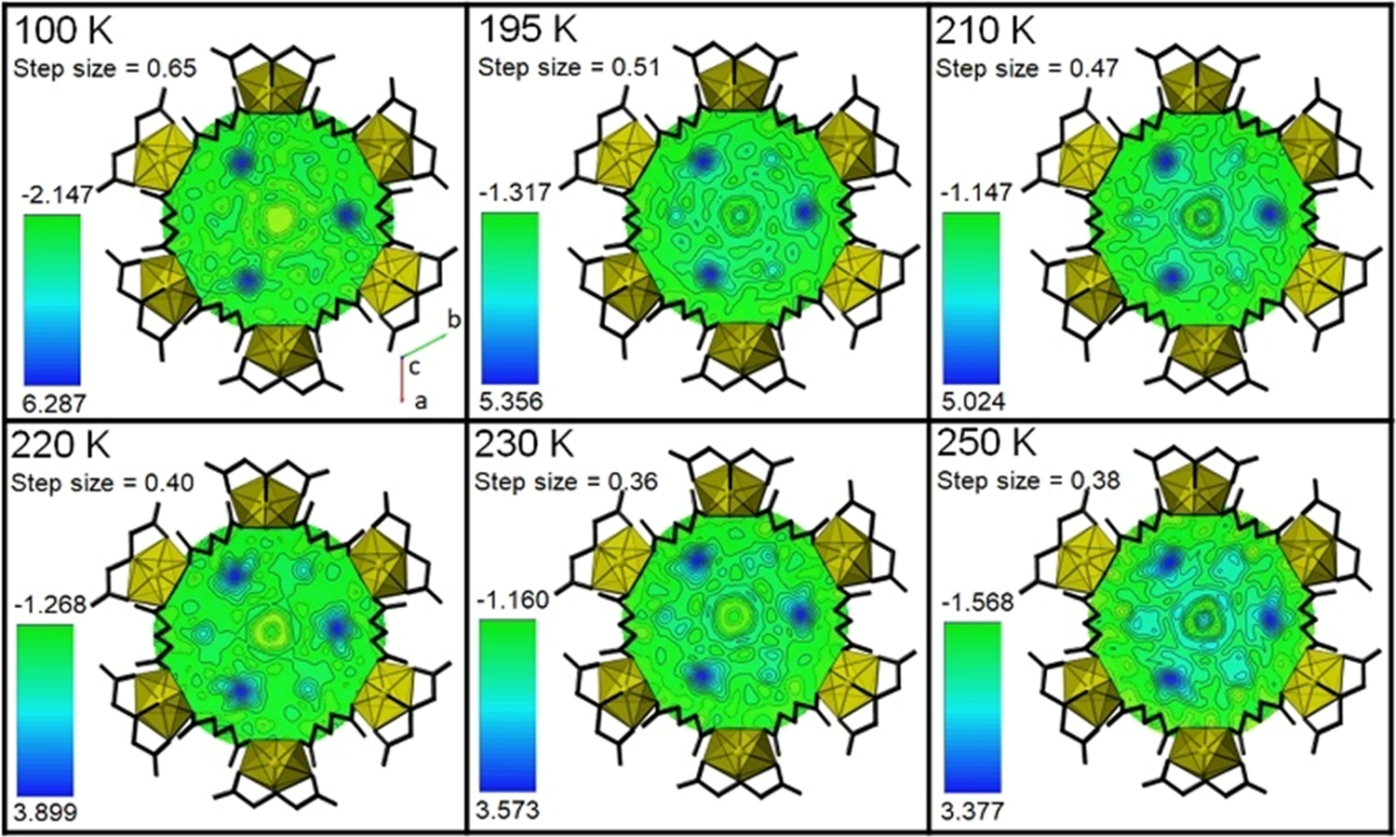
Electron density maps for the OW1 sites versus temperature with variable electron density scale within UMON. All maps are oriented in the same direction (looking down the *c* axis), and the U(VI) iminodiacetate nanotube has been placed on top of the electron density map for clarity in the orientation. The values for the electron density are given in e/A^3^.

**Figure 3:**
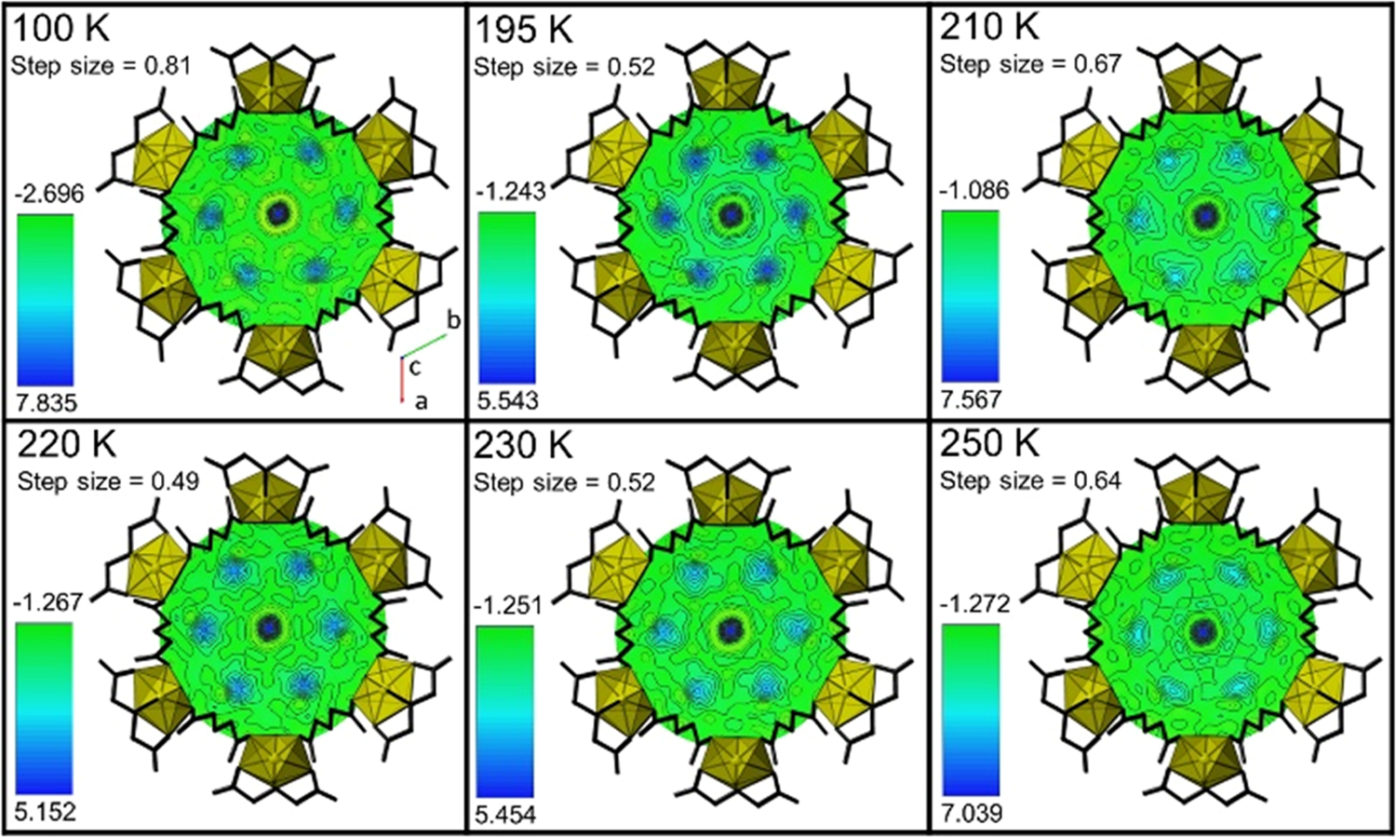
Electron density maps for the OW2 sites versus temperature with variable electron density scale within UMON. All maps are oriented in the same direction (looking down the *c* axis), and the U(VI) iminodiacetate nanotube has been placed on top of the electron density map for clarity in the orientation. The values for the electron density are given in e/A^3^.

To further evaluate the structural changes of the confined water molecules with varying temperature without imposing modeling constraints, we turned to the electron density maps created using the OLEX2 software.^56^ Positioning of the U atoms within the unit cell (Table S2)

were kept constant so that the maps represent the same orientation and depth. Two different depths were monitored (−1 and −7) along the (001) axis, which represented the planes associated with the previously described OW1 (Figure 2) and OW2 (Figure 3) sites, respectively. While all temperature data were analyzed, we have chosen six temperatures (100, 195, 210, 220, 230, and 250 K) to represent the observed changes in the water structure.

For the OW1 site (Figure 2), we initially observe three spherical regions of electron density at the maximum value on the −1 plane at 100 K. Three weak areas of electron density can be observed at the other corners of the hexameric ring, suggesting there are three water molecules within the plane and three below. Overall, this is consistent with the observed chair confirmation of the OW1 site that was modeled from the single-crystal X-ray diffraction data. This structural arrangement is maintained between 100 and 250 K, but there is diminishing electron density (from a maximum of 6.287 to 3.377 e/A^3^) within the discrete areas. In addition, the shape of the areas of high electron density becomes less spherical and more diffuse with an increasing temperature.

Turning to the OW2 site (Figure 3), we observe the nearly planar hexagonal ring between 100 and 250 K, with the addition of a small region at the central position of higher electron density. The presence of six areas of electron density at the same depth in the map indicates that the confirmation of this hexamer is planar. In addition, there is an area located at the center of the hexamer that exhibits a large residual electron density (OW3). This electron density can be observed during the full structural refinement of the UMONT material in this and previous studies but could not be successfully modeled with an O atom as the site will become nonpositive definite upon refinement. We also noted variability in the residual electron density of this site, with the original data published by Unruh et al. containing 1.77 e/A^3^ at the OW3 position.^50^ With an increase in temperature, the electron density in the OW2 site becomes more diffuse and decreases. However, the central OW3 site maintains a high electron density throughout the entire temperature range. Given the difficulties in modeling the OW3 site with the X-ray diffraction data, we then turned to neutron diffraction to confirm the presence of an atom at this central position.

### Confirmation of Structural Features Using Single-Crystal Neutron Diffraction

**Figure 4:**
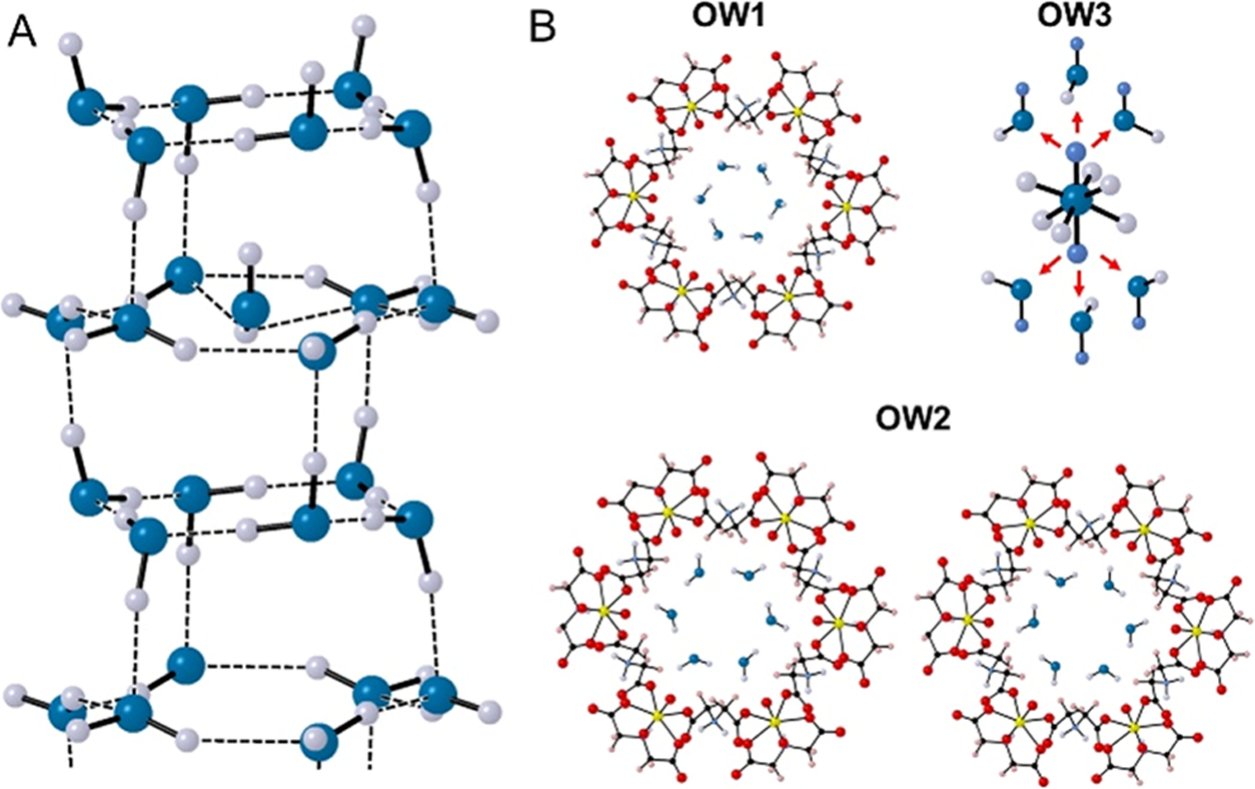
Single-crystal neutron diffraction was used to confirm the water and hydrogen-bonding network for the water confined within the UMONT material. (A) The ice-like array is confirmed, and a central water site was observed with 1/12th occupancy. (B) OW1 exhibited an ordered arrangement for the hydrogen atoms, but multiple split sites could be observed.

Single-crystal neutron diffraction data was collected at one temperature (100 K), and the nature of the OW1 and OW2 positions was again confirmed using this technique. OW1 is observed as a more ordered hexagonal ring with a chair conformation, whereas the hexagonal ring for OW2 is in a more planar hexameric configuration (Figure 4a). In addition, there was evidence to support the placement of an O atom in the center of the OW2 ring, although the site (OW3) was modeled as 1/12th occupied to obtain a reasonable displacement value (0.10202).

Neutron diffraction was also utilized to confirm hydrogen bonding within the structural topology (Figure 4b). Overall, the hydrogen positions for OW1 are identical to those we have previously reported, and the hydrogen bonding interactions occur solely with neighboring water molecules. For OW2, one D atom interacts with the neighboring water molecule within the planar ring and the second participates in deuterium-bonding interactions with the O atom on the carboxylate. There are two orientations that the OW2 can take, which leads to split positions. In both cases, the deuterium-to-donor distances between the neighboring water molecules were 2.328 and 2.220 Å and the donor-to-acceptor angle is 135.4°. Similar deuterium distances to the carboxylate O atoms (O3 and O6) were observed at 2.118 and 2.230 Å, but the bond angles were much closer to linear (175.1 and 174.0°).

The third partially occupied OW3 site located at the center of the planar hexagonal ring was also disordered, with six different orientations of the water molecule within this region. The equatorial deuterium atoms associated with the OW3 can hydrogen bond with the lone pairs on the OW2 atoms with a D···O distance of 2.300 Å, and the bond angles are unrealistic at 130.0°. The equatorial positions associated with the OW3 site are too far from the OW1 site to engage in hydrogen bonding, but the axial positions for this site may engage in weak interactions with a hydrogen bond distance of 3.361 Å and an angle of 163.1°.

Also notable with the OW3 site is that the axial positions needed to be modeled as partially occupied with hydrogen atoms instead of deuterium, suggesting that isotope exchange took place during the synthesis of the material. At this point, we do want to note that our recent work has indicated high selectivity of the UMONT material for H_2_O over HDO or D_2_O, so the presence of D_2_O within the nano porous channels would not be favored.^54^ However, it is important to emphasize the differences between these two studies, as our initial work looked at dehydrated UMONT that was exposed to D_2_O vapor, and the current study utilized deuterated solvent and reactants to create the crystalline material for neutron diffraction. In addition, the crystallization of the deuterated UMONT resulted in smaller crystals and lower yields, most likely because of the difference between deuterium and hydrogen bonding networks. Finally, the selectivity of this system for hydrogen is again displayed as we do see there is partial occupancy of hydrogen in the channel sites which is likely caused by trace levels in the deuterated system.

Overall, the variable-temperature X-ray and low-temperature neutron diffraction data provide key information for understanding the nanoconfined water structure. First, we confirmed that the hexagonal water structure occurred in two configurations (chair and planar motifs) and identified a new partially occupied water molecule in the center of the OW2 ring. Removing the modeling constraints imposed by the symmetry was key to clearly delineating the differences in the OW1 and OW2 hexagonal ring structures. Previous computational analysis of hexagonal ice within single-walled carbon nanotubes indicated the formation of chair confirmation throughout the nanochannel,^62^ but planar modes have previously been observed on surfaces.^63,64^

The presence of the central OW3 water has been previously identified by computational efforts evaluating water confined within single-walled carbon nanotubes but has not been experimentally characterized in these systems. Several groups have used molecular dynamic simulations to evaluate carbon nanotubes with subtle differences in diameter.^65−68^ Moid et al. have evaluated changes in the confined water structure for single-walled carbon nanotubes with a range of diameters at 1 atm of pressure.^65^ They observed a hexagonal ice channel without a central water site for a diameter of 1.22 nm at temperatures up to 250 K. However, when the diameter of the nanotube was changed to 1.36 nm then an eight-membered water ring with an additional water located in the middle of the channel can be observed for this system.^65^ In addition, Mochizuki and Koga found that at high pressures (>1 GPa), carbon nanotubes with a diameter of 1.11 nm possessed a “filled ice structure” where there was an additional water molecule present at the center of the hexagonal ring. Increasing the diameter of the nanotube to 1.23 nm resulted in less filling of the central site and a diameter of 1.25 nm resulted in an empty hexagonal ice-like array.^66^ In our case, the diameter of our nanotube (1.18 nm) fits within the theoretical value for observing hexagonal water rings, but we observed partial filling of this position at atmospheric pressure compared to values >1 GPa that were utilized in the previous study. The variability in the electron density in the OW3 site is likely due to the exact hydration of the UMONT material, with lower relative humidity values decreasing the overall occupancy in this site.

### Analysis of H_2_O Motion Using NMR Spectroscopy

**Figure 5:**
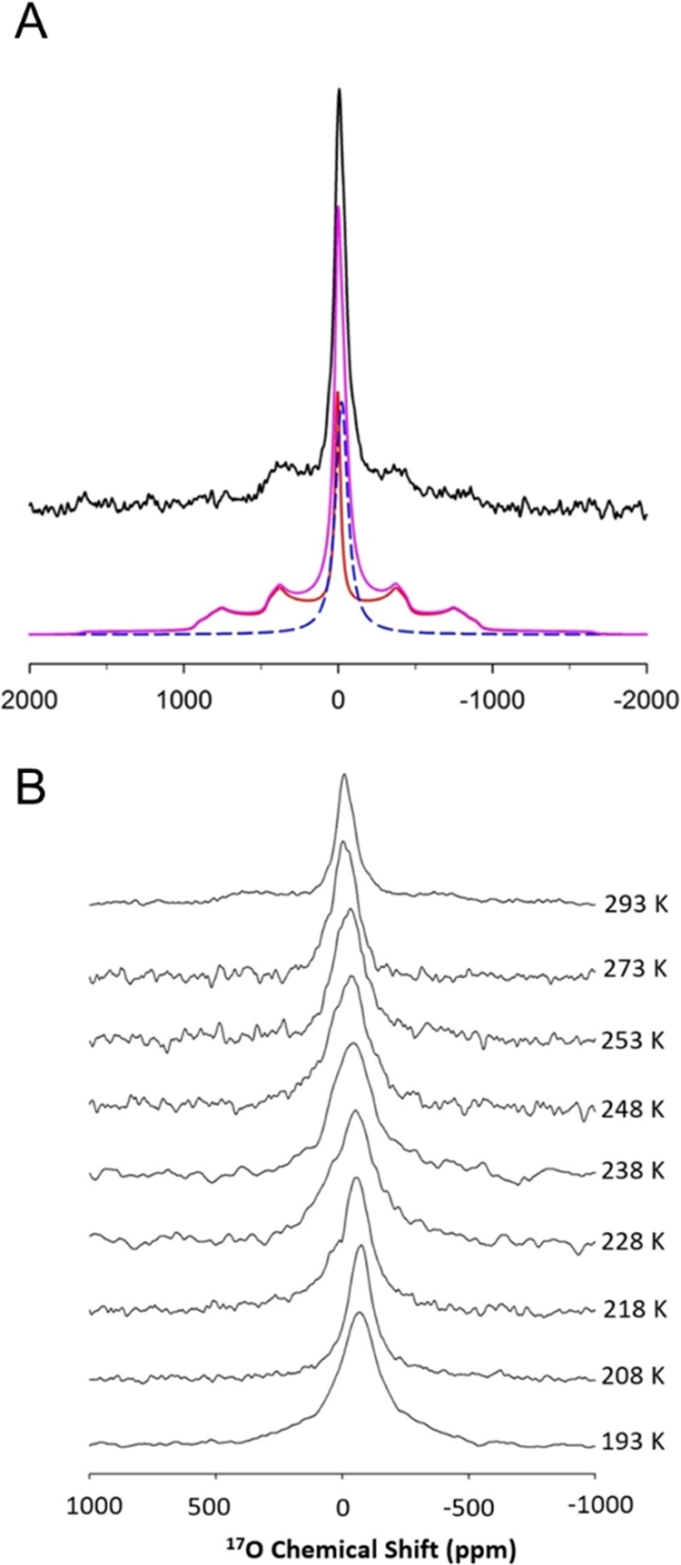
Static ^17^O NMR spectra for the nanoconfined water within the UMONT channel. (A) Spectrum obtained at 293 K with Lorentzian and quadruple line shape analysis. Simulation parameters include a Lorentzian peak width of 6440 Hz and a chemical shift of −23.4 ppm. For the signal with the quadruple pattern, the simulation parameters are CQ = 400 kHz, line broadening = 2830 Hz, chemical shift = 2.8 ppm, and etaQ = 0.1. (B) Changes in the ^17^O NMR signal with temperature.

While diffraction techniques provide information on the structural characteristics of the nanoconfined water with varying temperatures, we turn to NMR to evaluate the phase behavior. The static ^17^O NMR spectrum of the UMONT material with nanoconfined H_2_^17^O at room temperature contains a peak at approximately 0 ppm and two pairs of singularities that are typical satellite transitions of *I* = 5/2 nuclei. This line shape can be simulated into two components: a signal with Lorentzian line shape and one with a quadruple pattern. The modeling result is displayed in Figure 5a. The component with a Lorentzian line shape indicates the presence of mobile water molecules. On the other hand, the signal with quadruple pattern and low-asymmetry parameter (etaQ = 0.1) clearly suggests the structural environment of ^17^O in a rigid lattice with nearly uniaxial symmetry. The ^17^O NMR observation is consistent with the X-ray diffraction (XRD) result. Line shape simulations also indicate that the intensity ratio of the two signals is about 33/67 for mobile/rigid water contained within the channels.

**Figure 6:**
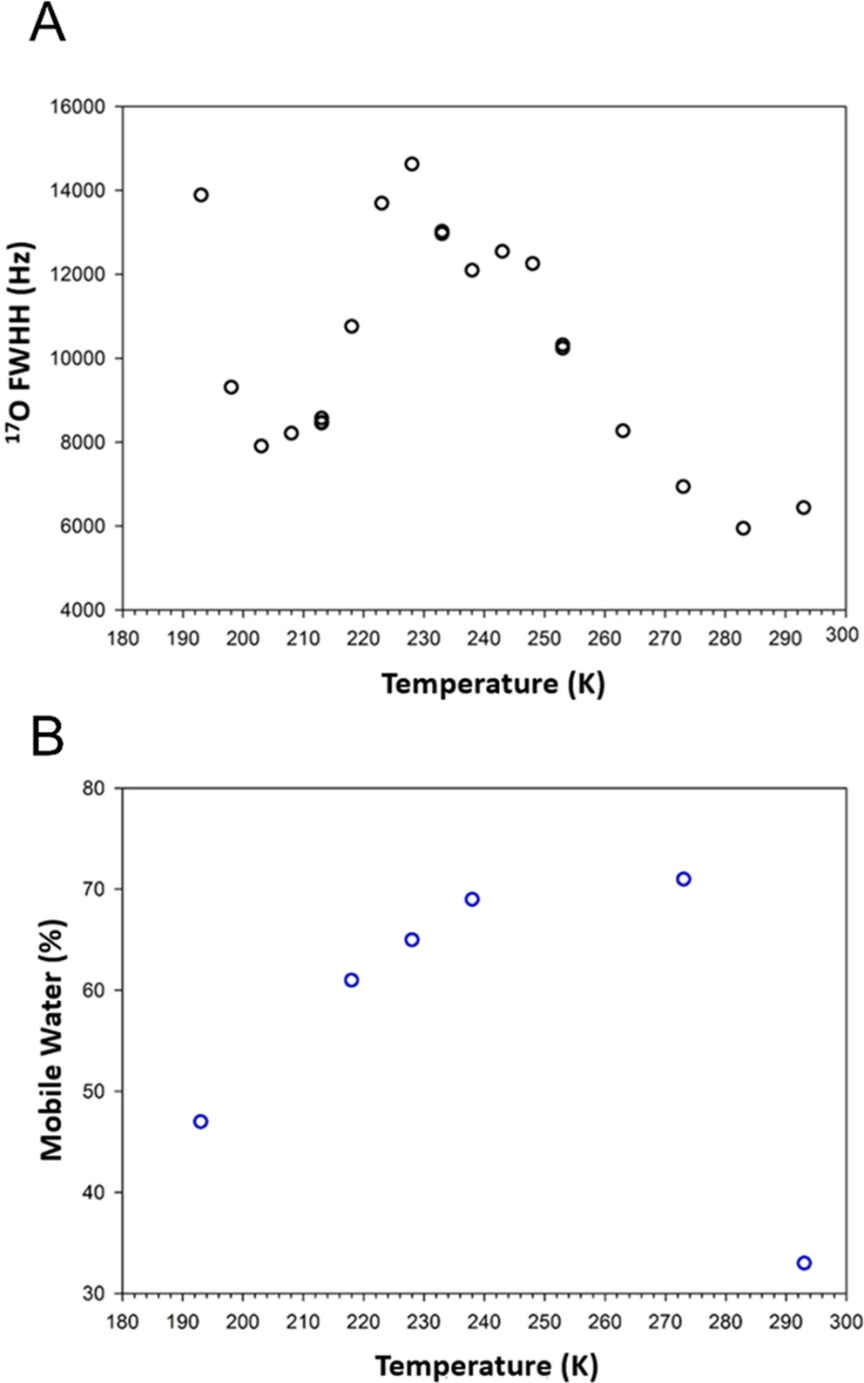
(A) ^17^O NMR full width at half-height (fwhh) of the modeled line shape analysis for water confined within the UMONT with variable temperature. (B) Modeling of the percent mobile water for the hydrated UMONT material versus temperature.

With a decrease in temperature, each component of the line shape increases with a decrease in temperature (Figure 5b). The Lorentzian-shaped signal becomes broader in width but maintains the overall line shape. However, the quadruple line broadens to the point that the singularities for the satellite transitions are very weak in intensity and distributed beyond the spectrum bandwidth. All spectra were simulated with two components, and we observed that the peak width of the central signal changed as a function of temperature (Figure 6a). This signal contains intensities from all of the transitions (central and two pairs of satellite transitions). These satellite transitions cannot be distinguished from the central transition; thus, they are all averaged into one Lorentzian peak due to the fast motion of the water molecules. When temperature decreases, the water motion becomes restricted and the averaging/exchange rate becomes slower, which broadens the NMR signal, particularly in the temperature range between 293 and 233 K. Within these broad signals, the averaging effect of the satellite transitions no longer dominate the line shape, and the satellite transitions are separated from the central transition. This happens when the water becomes rigid and depicts the transition from more liquid-like to solid-like behavior. The signal now contains only central transition and therefore becomes narrower in width. In the case of the hydrated UMONT material, that initially occurs at 233 K and continues to progress until 208 K. At lower temperatures (between 208 and 193 K), the signal for the central transition becomes broader with decreasing temperature due to dipolar interaction from more restricted hydrogen atoms.

With the line shape simulations, the relative ratios of the rigid versus mobile water can again be estimated with a variable temperature (Figure 6b). The amount of mobile water is calculated at 70% at 275 K and remains close to this value until 240 K. With decreasing temperature, the amount of mobile water continues to decrease as well. The lowest temperature collected in these experiments was at 195 K and the calculated mobile water was 47% at this point. If we assume a linear response to the reduction of mobile water with decreasing temperature,^67^ then the amount of mobile water will reach zero at 98 K.

Two-component line shape analysis has also been observed for water confined within single-walled carbon nanotubes, but there are some significant differences observed for the hydrated UMONT system.^68^ Ghosh et al. evaluated carbon nanotubes with a diameter of 1.2 nm using ^1^H NMR and found that there were two spectral components to the line shape that appeared at 242 K, but the mobile signal disappeared at 217 K.^69^ They attributed this behavior to the structural features of the water channel, suggesting that there was a water tube with similarities to the hexagonal rings observed in UMONT and a second water channel in the middle of the pore. Ghosh et al. suggested that the water-ice transition temperature for the water tube occurred at 242 K and then the central water remained liquid-like until 217 K.^69^ Similar phenomena are also observed for carbon nanotubes of slightly larger diameters, with subtle differences occurring during the water-ice transition.^67,70^ For the UMONT material, the ice-like behavior is present at 293 K and then two spectral components are observed throughout the temperature range studied. This suggests that the completion of the water-ice transition occurs above room temperature or does not occur before dehydration of the UMON occurs at 335 K.^50^ Therefore, we turned to differential scanning calorimetry to evaluate the thermal behavior of the water within the UMONT material.

### Differential Scanning Calorimetry

**Figure 7:**
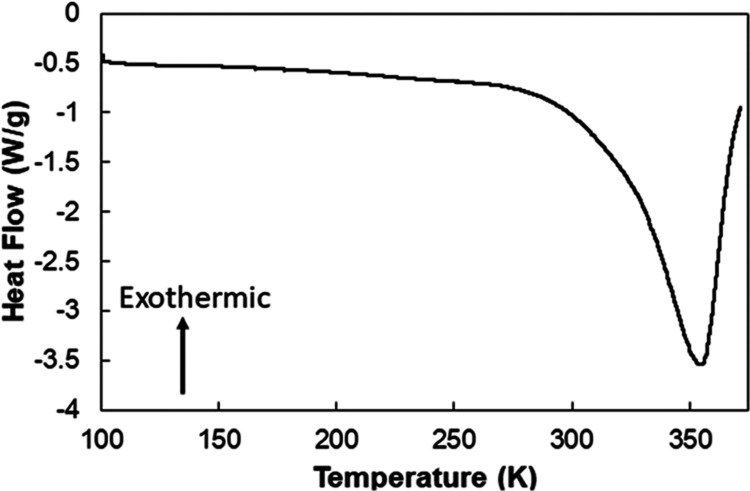
Differential scanning calorimetry (DSC) data for UMONT between 100 and 375 K at a scan rate of 5°/min.

We initially evaluated the thermodynamics of the hydrated UMONT material from 100 to 375 K using Differential Scanning Calorimetry (DSC) (Figure 7) and found no evidence of any significant phase changes in the range from 100 to 293 K. There is an endothermic feature that occurs between 293 and 375 K that corresponds to the removal of the water from the UMONT channels. While the lowest temperature we can obtain on our instrument is 100 K, we do not see any evidence of a peak onset in this area, nor do we see evidence of a peak at higher temperatures that would correspond to the final melt temperature.

The absence of a phase transition for the UMONT material is again different than what has previously been observed experimentally for water under nanoconfinement, but there is theoretical evidence for carbon nanotubes. DSC measurements performed by Kyakuno et al. on carbon nanotubes with diameters between 1.7 and 2 nm observed a clear endothermic peak between 200 and 225 K that they attributed to the water-ice phase transition.^27^ The overall diameter of the carbon nanotubes plays a significant role in the exact melting point, which has been reported to range between 200 and 300 K.^21,71,72^ In addition, Koga evaluated the freezing of liquids in quasi-1-D pores with 1 to 3 nm diameters and found that the phase change of the water in these systems occurs gradually.^73^ However, Mukherjee et al. also performed molecular dynamic simulations of carbon nanotubes with a diameter of 0.8 nm that contain single chains of water molecules and they found that the confined water behaves like a solid up to 300 K.^74^ In this case, the spatial ordering of the water molecules is likely due to the strong hydrogen bonding that occurs along the length of the channel. Simulations on carbon nanotubes with a similar diameter to that of UMONT are not predicted to behave in a similar manner as an abrupt freezing point is expected to occur at ∼250 K.^75^

Lack of a phase transition in the DSC is similar to what is reported by Jahnert et al. for hydrophilic, cylindrical silica nanopores that are less than 2.6 nm in diameter.^76^ In this study, DSC was used to determine the melting point for silica nanopores between 3.0 and 4.4 nm, but no signal was observed for the smaller pore size. They turned to NMR spectroscopy to further analyze these materials and determined that the melting point (first evidence of mobile water) for the 2.6 nm diameter silica pore was 218 K and the width of the phase transition was 18 K (up to 236 K). Based upon the linear regression of the mobile water obtained from NMR spectroscopy,^67^ we can suggest melting point begins at 98 K, but again we see no evidence of the phase transition before the water is removed from the material at 293–360 K.Combining the confinement effects of the pore diameter and the hybrid nature of the UMONT materials likely leads to the observed behavior of the water. Taking a closer look at the water interactions, we note that the OW1 site engages in hydrogen bonding with only other water molecules within the channel, but the OW2 site has a weak interaction with the interior walls of the UMONT material. This weak interaction has been previously measured at ∼7 kJ/mol,^77^ which is closer in energy to what would be expected for a hydrophobic channel wall than those observed for hydrophilic surfaces. These weak interactions are likely enough to stabilize a portion of the water molecules within the channel and the strong hydrogen bonding network between the water molecules, which is different from the complete hydrophobicity that can be observed for single-walled carbon nanotubes. However, X-ray and neutron diffraction suggest that OW1 is the more ordered ice-like form, and OW2 has more anisotropic electron density and positional disorder in the solid-state structure. It is possible that OW2 has two preferential hydrogen bonding sites on the interior walls of the channel that would lead to positional disorder in the crystal structure.

## Conclusions

We have provided a detailed analysis of the structure and mobility of the nanoconfined water within the UMONT structure materials, and the results highlight the importance of surface chemistry on the overall behavior of water within these pore spaces. Based upon the temperature-dependent single-crystal X-ray and neutron diffraction studies of the UMONT compound, we provided the first experimental evidence of a filled hexagonal ice structure within a 1D nanochannel under ambient pressures. Neutron diffraction data confirmed the presence of a central OW3 site, and we note that the occupancy of this position can vary on the basis of the hydration state of the material. ^17^O NMR suggests that the onset of melting for the water in the UMONT channels likely occurs at 98 K and we observe the presence of ice-like water up to 293 K, indicating that the complete ice–water transition does not occur before dehydration of the material. This observation was supported by the lack of a signal in the DSC curve between 100 and 360 K. This behavior differs significantly from hydrophobic single-walled carbon nanotubes with the same pore diameter, where the water-ice transition occurs between 217 and 242 K.

Our work highlights the importance of exploring hybrid materials, which offer tunability in the placement of the hydrophobic and hydrophilic functional groups and precise controls over water structure and phase behavior. Based upon the observed difference between the UMONT material and carbon nanotubes, our results suggest that water confined in other hybrid materials may also display variations in the water structure and behavior. Water confined within MONT materials already displays unique water topologies that are likely due to the nature of the functional groups that are present on the interior channel wall.^43^ Rationally designing materials with variable placement of hydrophobicity along the interior pore walls can lead to a difference in the water structure and influence the ferroelectric behavior or proton conductivity for these compounds. However, there is very limited information regarding the behavior of the water within porous hybrid material, and systematically evaluating MONT materials to compare to similarly sized carbon nanotubes will offer more insights into the influence of pore diameter versus channel chemistry. Future efforts will explore water structure and mobility in hybrid materials with variable hydrophobic and hydrophilic features to further delineate the structure–function relationships for water under nanoconfinement.

## Supporting Information Available

Crystallographic parameters for UMON at different temperatures (including modeling of water molecules) (Table S1); uranium atom fractional coordinate over the temperature change (Table S2); and electron density map at OW1 and OW2 positions at 270 K (Figure S1) (PDF)
Crystallographic data for all temperatures (CIF)
UMONT neutron (CIF)

## Supplementary Material

la3c02786_si_001.pdf

la3c02786_si_002.cif

la3c02786_si_003.cif
