# Supplementary material for: Characterization of Water Structure and Phase Behavior within Metal–Organic Nanotubes
Source: Langmuir. 2023 Dec 11;39(51):18899–908. doi: 10.1021/acs.langmuir.3c02786 (PMC10753883; doi:10.1021/acs.langmuir.3c02786)
Supplement: Supplementary file 1 — la3c02786_si_001.pdf [file la3c02786_si_001.pdf]

Electronic Supplementary Information

Characterization of Water Structure and Phase Behavior Within Metal Organic Nanotubes

Tiron H. L. Jahinge,[a] Maurice K. Payne, [a] Daniel K. Unruh, [a] Ashini S. Jayasinghe, [a] Ping Yu,[b]

and Tori Z. Forbes [a]*

a. Department of Chemistry, University of Iowa, Iowa City, IA 52242.

b. Nuclear Magnetic Resonance Facility, University of California, Davis, Davis, CA 95616.

Table of Contents

Table S1. X-ray Crystallographic parameters for UMON at different temperatures (including modelling of water molecules).

Table S2. Uranium atom fractional coordinate over the temperature change based upon X-ray diffraction data.

Figure S1. Electron density map of X-ry diffraction data at (A) OW1 and (B) OW2 positions at 270K.

Figure S2. OW1 bond distances and angles obtained using neutron diffraction.

Figure S3. OW2 bond distances and angles obtained using neutron diffraction.

Table S1. Crystallographic parameters for UMON at different temperatures (including modelling of water molecules).

Temp (K)	R_1 (%)	R_{int} (%)	GOF	wR_2 (%)	Max Q peak	a (Å)	c (Å)	V ($Å^3$)
100	3.78	5.33	1.093	7.54	5.5	22.2935(7)	6.6090(3)	2844.61(18)
195	3.59	7.29	1.064	7.45	1.2	22.4050(6)	6.6040(2)	2870.96(14)
210	3.71	7.75	1.045	7.60	1.4	22.4339(6)	6.5980(3)	2875.76(17)
220	3.65	6.47	1.083	8.86	4.9	22.4549(5)	6.5957(2)	2880.14(12)
230	3.53	6.76	1.058	8.30	1.4	22.4784(5)	6.5920(2)	2884.55(12)
250	4.57	9.21	1.037	10.93	1.0	22.5614(9)	6.5936(3)	2906.60(2)
270	4.41	9.36	1.042	10.01	1.3	22.5295(11)	6.5845(5)	2894.40(3)

Table S2. Uranium atom fractional coordinate over the temperature change.

Temperature (K)	x	y	z
100	0.79357	0.17256	0.67118
195	0.79353	0.17286	0.66932
210	0.79353	0.17300	0.66896
220	0.79353	0.17318	0.66853
230	0.79354	0.17331	0.66833
250	0.79355	0.17349	0.66785
270	0.79393	0.17343	0.66813

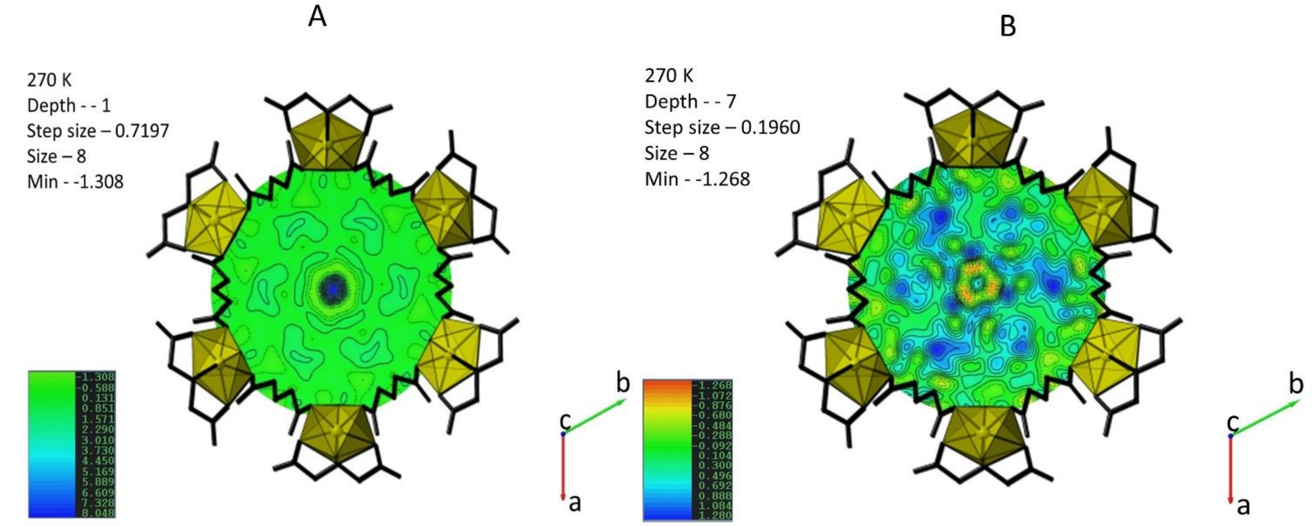

Figure S1. Electron density maps at (A) OW1 and (B) OW2 positions at 270K.

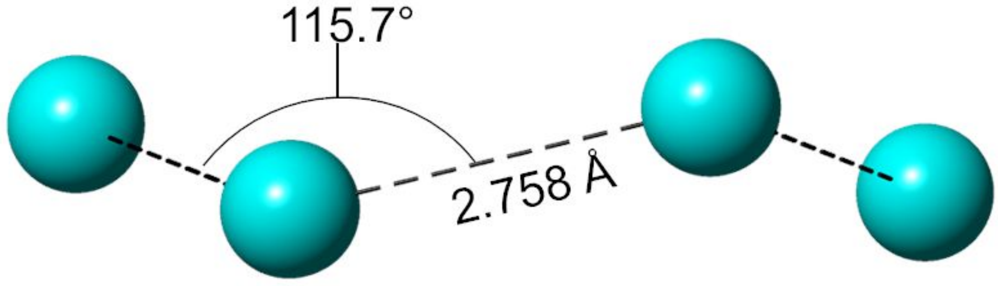

Figure S2. OW1 bond distances and angles obtained using neutron diffraction.

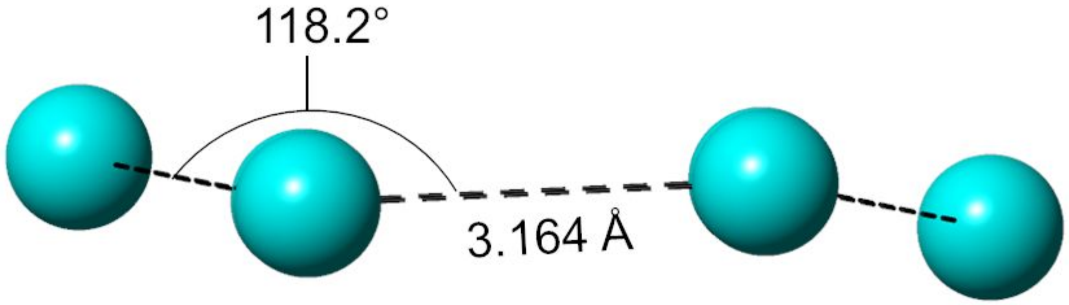

Figure S3. OW2 bond distances and angles obtained using neutron diffraction.

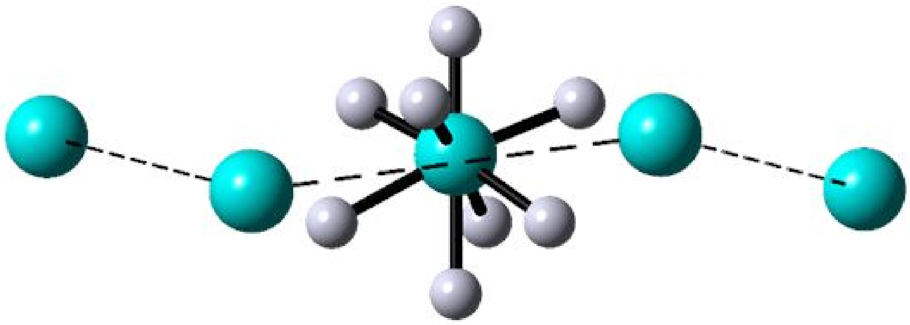

Figure S4. OW2 ring with OW3 water molecule located in the central position obtained with neutron diffraction. The gray atoms represent the different deuterium positions modeled from the diffraction data. The closest D3b-OW2 distance is 2.300 Å and the OW3-D3b-OW2 angle is 130°.
